# Identification, Biochemical Characterization, and In Vivo Detection of a Zn-Metalloprotease with Collagenase Activity from *Mannheimia haemolytica* A2

**DOI:** 10.3390/ijms25021289

**Published:** 2024-01-20

**Authors:** Gerardo Ramírez-Rico, Moises Martinez-Castillo, Lucero Ruiz-Mazón, Erika Patricia Meneses-Romero, José Arturo Flores Palacios, Efrén Díaz-Aparicio, Erasmo Negrete Abascal, Mireya de la Garza

**Affiliations:** 1 Faculty of Professional Studies Cuautitlan, Autonomous National University of Mexico (UNAM), Mexico City 54714, Mexico; garmvz@gmail.com; 2 Department of Cell Biology, Center for Research and Advanced Studies, Mexico City 07360, Mexico; luceroruizmazon@gmail.com; 3 Liver, Pancreas and Motility Laboratory, Unit of Research in Experimental Medicine, School of Medicine, Autonomous National University of Mexico (UNAM), Mexico City 06726, Mexico; mpcastillo@comunidad.unam.mx; 4 Laboratory of Proteomics, Biotechnology Institute IBT-UNAM, Cuernavaca 62210, Mexico; erika.meneses@ibt.unam.mx; 5 Hayko Scientific Laboratories, Mexico City 14420, Mexico; arturo.flores@haykoscientific.com.mx; 6 National Center for Disciplinary Research in Animal Health and Safety, National Institute of Forestry, Agricultural and Livestock Research (INIFAP), Mexico City 05110, Mexico; 7 Faculty of Professional Studies Iztacala, Autonomous National University of Mexico (UNAM), Mexico City 54090, Mexico; negretee@yahoo.com

**Keywords:** *M. haemolytica*, metalloproteases, zymograms, collagen

## Abstract

Respiratory diseases in ruminants are a main cause of economic losses to farmers worldwide. Approximately 25% of ruminants experience at least one episode of respiratory disease during the first year of life. *Mannheimia haemolytica* is the main etiological bacterial agent in the ruminant respiratory disease complex. *M. haemolytica* can secrete several virulence factors, such as leukotoxin, lipopolysaccharide, and proteases, that can be targeted to treat infections. At present, little information has been reported on the secretion of *M. haemolytica* A2 proteases and their host protein targets. Here, we obtained evidence that *M. haemolytica* A2 proteases promote the degradation of hemoglobin, holo-lactoferrin, albumin, and fibrinogen. Additionally, we performed biochemical characterization for a specific 110 kDa Zn-dependent metalloprotease (110-Mh metalloprotease). This metalloprotease was purified through ion exchange chromatography and characterized using denaturing and chaotropic agents and through zymography assays. Furthermore, mass spectrometry identification and 3D modeling were performed. Then, antibodies against the 110 kDa-Mh metalloprotease were produced, which achieved great inhibition of proteolytic activity. Finally, the antibodies were used to perform immunohistochemical tests on postmortem lung samples from sheep with suggestive histology data of pneumonic mannheimiosis. Taken together, our results strongly suggest that the 110-Mh metalloprotease participates as a virulence mechanism that promotes damage to host tissues.

## 1. Introduction

*Mannheimia haemolytica* is a normal inhabitant of upper respiratory tracts in ruminants and maintains a commensal relationship with its host. However, when the host is under stress and subject to certain other biological conditions, *M. haemolytica* can act as a ruminant-specific pathogen that is primarily associated with respiratory disease. In fact, *M. haemolytica* is the most important bacterial pathogen in the bovine respiratory disease complex [1]. It has been reported that lambs and sheep are mainly infected by *M. haemolytica* serotype A2 and that bovines are mainly infected by serotype A1 [2,3]. In sheep, *M. haemolytica* has been associated with pneumonic pasteurellosis (mannheimiosis), a complex disease that involves host factors and other infectious agents, which cause significant economic losses due to mortality, reduction in weight gain, treatment costs and penalties, or confiscations in slaughterhouses [4,5,6]. *M. haemolytica* promotes alteration in alveolar epithelium and acts as an opportunistic bacterium during infections caused by viruses, such as parainfluenza-3, herpesvirus, respiratory syncytial virus, adenovirus, and reovirus.

Currently, methods to control ruminant respiratory disease include aggressive antimicrobial therapies, including administering antibiotic treatments to cattle upon arrival to farms (metaphylaxis) [7,8]. Anti-*M. haemolytica* vaccines have reached clinical trials, but their effectiveness was low or limited. Thus, no commercial vaccine is available for this disease, possibly explaining why pneumonic respiratory complex disease remains the major cause of cattle morbidity, mortality, and reduced production, which costs the United States cattle industry approximately USD 1 billion per year [9]. Furthermore, the severity of clinical signs can vary from an inapparent illness to a rapidly fatal disease. The most common tissue features in lung lesions include the extensive infiltration of neutrophils and macrophages, the exudation of fibrin into the airways, and occasionally hemorrhages [10,11].

*M. haemolytica* has several secreted virulence factors, such as a potent leukotoxin and proteases that can be secreted during the infection process [12,13,14,15,16,17,18]. Proteases are enzymes that catalyze the specific hydrolysis of one or more peptidyl bonds, and they are also called proteinases or peptidases [19]. In *M. haemolytica* A2, a 35 kDa Zn-metalloglycoprotease that acts on the sialoglycoproteins of leukocytes and lung epithelium has been described [20]. On the other hand, the proteolytic activities from culture supernatants (CS) and outer membrane vesicles (OMVs) of *M. haemolytica* A2 were identified and characterized by our group as cysteine and metalloproteases [17]. The objective of this work was the identification and purification of a 110 Zn-metalloprotease from *M. haemolytica* A2. We obtained evidence that 250 and >250 kDa proteases from the CS of *M. haemolytica* A2 promote the degradation of bovine gelatin, hemoglobin, holo-lactoferrin (holo-Lf), albumin, and porcine fibrinogen; these activities were found through zymography. Furthermore, a biochemical characterization of the 110 kDa Zn-metalloprotease showed activity against bovine collagen. The antibodies against the metalloprotease inhibited its activity and detected the protease in the sheep lungs with pneumonic mannheimiosis.

## 2. Results

### 2.1. Zymography of Proteins Precipitated with Ammonium Sulfate (PpAs) from the CS of M. haemolytica A2

**Figure 1:**
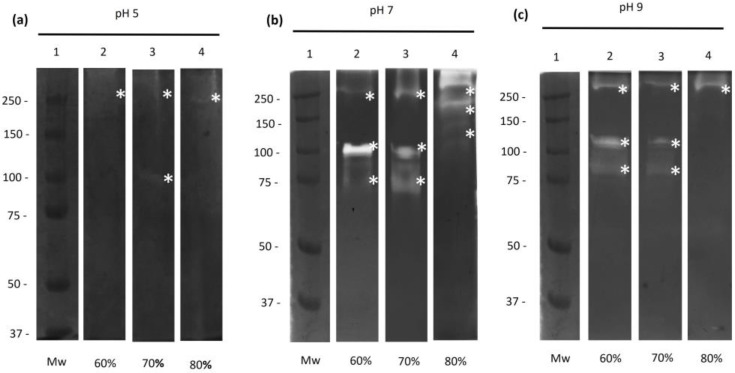
Zymograms of polyacrylamide gels copolymerized with bovine gelatin. Twenty micrograms of proteins precipitated with ammonium sulfate (PpAs) from the culture supernatant (CS) of M. haemolytica were loaded on 10% polyacrylamide gels copolymerized with 0.2% bovine gelatin. The following buffers were tested to determine the pH of activation: (**a**) pH 5.0, (**b**) pH 7.0, and (**c**) pH 9.0. Mw, molecular weight markers (Lane 1); fraction with 60% (Lane 2), 70% (Lane 3), or 80% (Lane 4) of (NH_4_)_2_SO_4_. Proteases are marked with an *. Representative results from three independent sample trials are shown.

The proteolytic degradation profiles of PpAs from the CS were analyzed through zymograms of acrylamide gels copolymerized with bovine gelatin at different pH values. Proteolytic activities showed lower activity at pH 5.0 (Figure 1a) than pH 7.0 (Figure 1b) and pH 9.0 (Figure 1c). Proteases of 75, 110, and 250 kDa were found in the fractions precipitated with 60% and 70% (NH_4_)_2_SO_4_ (Figure 1a–c, Lanes 2 and 3). In contrast, in the 80% fraction, only 250 kDa activity was observed at all pH values tested (Figure 1a–c, Lane 4). At pH 9.0, two extra activities were observed at 130 and 150 kDa. At a physiological pH of 7.0, the 110 kDa protease showed the strongest activity in the fraction containing 60% (NH_4_)_2_SO_4_. Thus, our efforts were focused on characterizing this protease.

### 2.2. Determination of the Proteolytic Activity of 60% PpAs from M. haemolytica A2 against Several Mammalian Proteins

**Figure 2:**
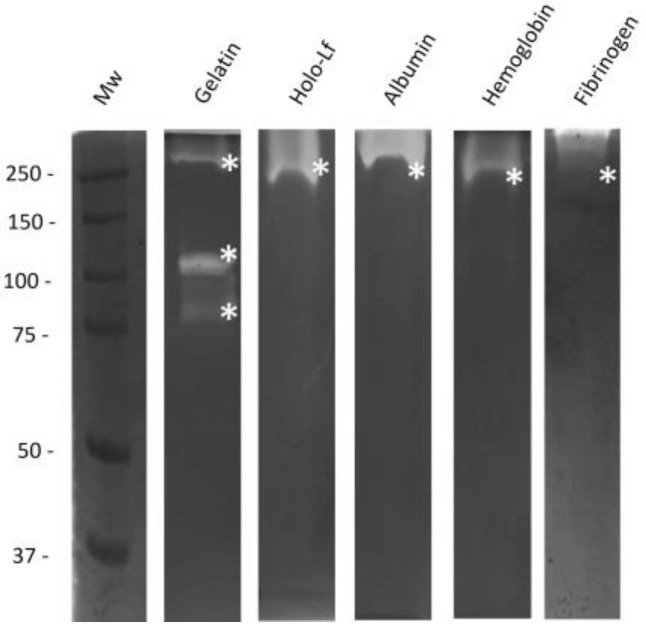
Zymograms of the fraction with 60% of (NH4)_2_SO_4_ from the CS of *M. haemolytica* A2. Gels were copolymerized with 0.2% of bovine gelatin, holo-lactoferrin (holo-Lf), albumin, hemoglobin, or porcine fibrinogen. They were incubated overnight with activation buffer pH 7.0. Proteases are marked with an *. Representative results from three independent sample trials are shown.

Since the 110 kDa-Mh protease showed great activity at physiological pH in gelatin (Figure 1b, Lane 2), we evaluated its ability to degrade other host proteins. For this, zymograms were performed using other substrates, such as holo-Lf, albumin, hemoglobin, and fibrinogen, with the PpAs fraction at 60%. The results revealed that the 110 kDa-Mh protease could only degrade bovine gelatin, whereas the proteolytic activity of 250 kDa could promote the degradation of all substrata tested, making it a nonspecific protease (Figure 2). This result suggests that the 110 kDa-Mh protease could be a collagenase; thus, we purified the protease and tested it against bovine collagen type I.

### 2.3. The 110-kDa Mh Protease Was Purified through Cationic Exchange Chromatography

**Figure 3:**
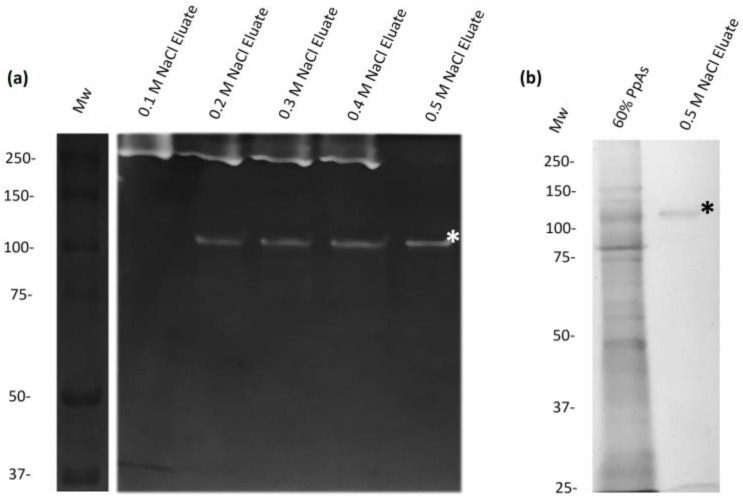
Purification of the 110 kDa-Mh protease by cationic exchange chromatography. Eluates evaluated in (**a**) zymograms of 10% gels copolymerized with 0.2% bovine gelatin with 0.1–0.5 M NaCl. The 110 kDa-Mh protease is observed in the 0.5 M NaCl eluate, is marked with *. (**b**) 10% SDS-PAGE with Coomassie blue stain. A band of 110 kDa, which coincides with the molecular weight of the purified proteolytic activity, is marked with *. Representative experiment of three independent samples.

Proteins precipitated with 60% of (NH_4_)_2_SO_4_ from CS were loaded onto previously equilibrated cationic exchange columns. The eluates were tested using zymograms copolymerized with bovine gelatin. The 110 kDa activity was separated from other proteolytic activities with 0.5 M NaCl (Figure 3a). The eluate of 0.1 to 0.4 M NaCl contained an activity of 250 kDa (Figure 3a). On the other hand, SDS-PAGE of the 0.5 M NaCl displayed a clear protein of 110 kDa, which correlates with proteolytic activity (Figure 3b).

### 2.4. The 110 kDa Protease of M. haemolytica A2 Is a Heat-Resistant Enzyme That Possesses Collagenolytic Activity

**Figure 4:**
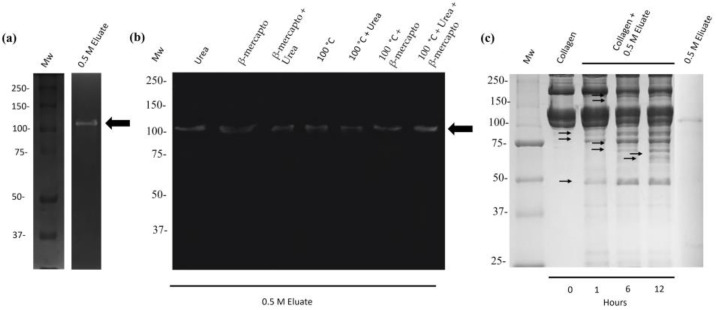
Degradation of type-I collagen by the 100 kDa-Mh protease. (**a**) The 0.5 M NaCl eluate was evaluated in zymograms of 10% polyacrylamide copolymerized with 0.2% bovine type-I collagen at pH 7.0 or (**b**) with different chaotropic and reducing denaturing agents. A proteolytic band is observed (black arrow). (**c**) The 10% SDS-PAGE was used to evaluate the cleavage and consequent formation of peptides derived from the α1, α2, and β chains, respectively (arrows). Representative experiment of three independent samples.

As the 110 kDa protease secreted by *M. haemolytica* A2 degraded bovine gelatin, we tested whether this protease could be a collagenase because gelatin is a derivative of collagen. First, a zymogram of 10% polyacrylamide copolymerized with 0.2% type 1 bovine collagen was carried out. Additionally, the interaction of different chaotropic denaturing and reducing agents on the protease purified by zymography was tested, as mentioned above. The results show that the 110 kDa-Mh protease can degrade collagen (Figure 4a) and was not affected by any chaotropic and reducing denaturing agents evaluated, as collagenase activity was always observed with a molecular weight of 110 kDa, even at 100 °C (Figure 4b); therefore, the protease is heat-resistant.

On the other hand, 10% SDS-PAGE was performed to identify the peptides derived from collagen after cleavage through the 110 kDa purified protease (Figure 4c). This enzyme specifically cleaved the α1 and α2 chains after the first hour of incubation with type-I bovine collagen, forming peptides of 90, 75, 73, 70, 64, 60, and 55 kDa. However, it also caused the β chain to undergo hydrolysis at the sixth hour of incubation, resulting in peptides of 190 and 180 kDa (Figure 4c).

### 2.5. The 110 kDa Protease with Collagenase Activity Secreted by M. haemolytica A2 Belongs to the Zn-Dependent Family of Metalloproteases

**Figure 5:**
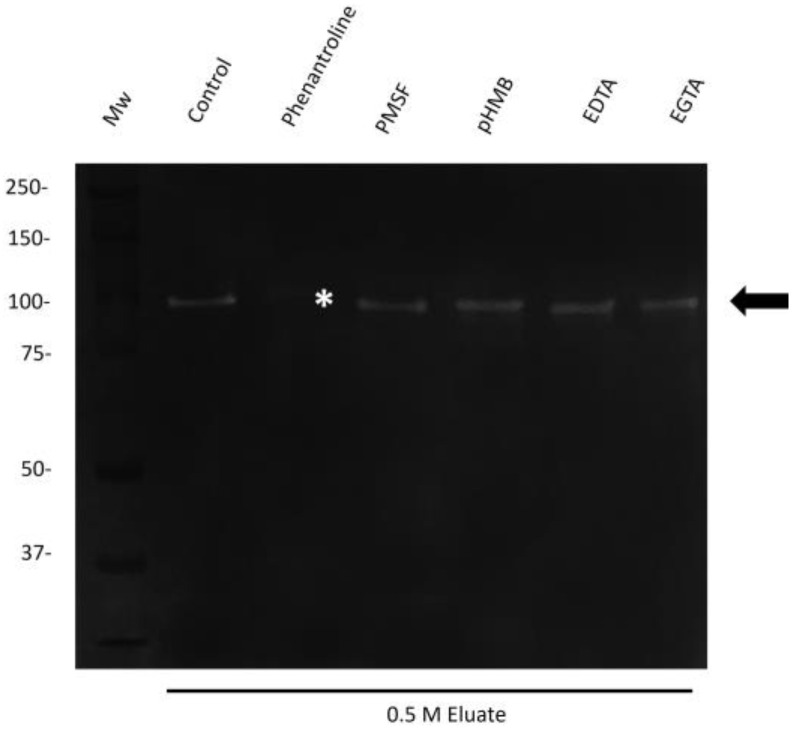
Inhibition of the 110 kDa-Mh protease with collagenase activity. The 110 kDa protease incubated with different protease inhibitors was evaluated for inhibition in zymograms of 10% gels copolymerized with 0.2% type-I collagen. Protease is indicated with a black arrow. Inhibition of the 110 kDa-Mh protease was observed only with phenanthroline, marked with *. Representative results of three independent sample trials.

After the proteolytic activity of the 110 kDa, the protease was partially characterized; knowing that the protease is substrate-specific (collagen), inhibition tests were performed to determine the collagenolytic activity. To perform this, the 0.5 M NaCl eluate was incubated with inhibitors of metallo, cysteine, and serine proteases. The inhibition results showed that collagenolytic activity was completely abolished with phenanthroline, a specific inhibitor of Zn-dependent metalloproteases (Figure 5).

### 2.6. The 110 kDa-Mh Protease Was Confirmed as a Zn-Dependent Metalloprotease through Mass Spectrometry

Once the characterization of the 110 kDa Zn-dependent metalloprotease with collagenase activity (110-Mh metalloprotease) was performed, the bands of proteolytic activity from zymograms and the 110 kDa protein bands from SDS-PAGE were analyzed through mass spectrometry LC-MS/MS. The analysis revealed approximately 20 fingerprints with two identified proteins corresponding to proteases; however, only one shared molecular weight close to 110.3 kDa, protease 3 OS, with accession number A0A249A431. This protease coincides with the results obtained from our characterization of the 110-Mh metalloprotease, belonging to the family of Zn-dependent metalloproteases and within the *M. haemolytica* genome. Furthermore, a good score (44.3%) and good coverage (37.1%) were obtained.

### 2.7. Prediction of the 3D Structure and Catalytic Site of the 110-Mh Metalloprotease

**Figure 6:**
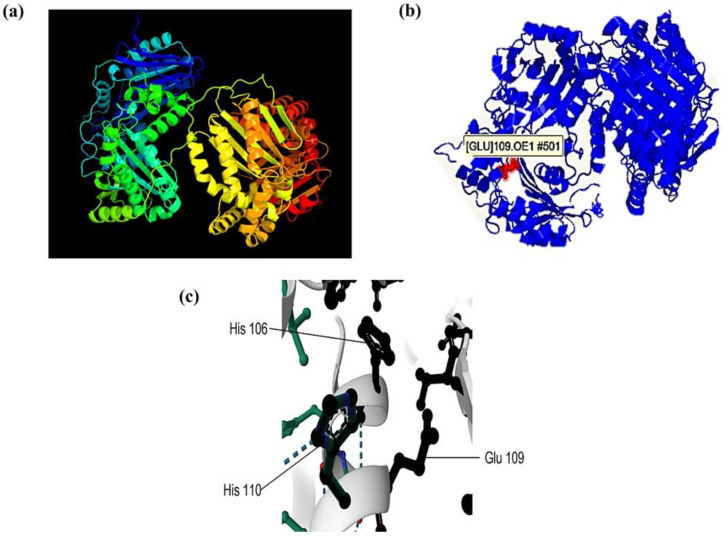
Three-dimensional modeling and catalytic site prediction of the 110-Mh metalloprotease. (**a**) Prediction made by Phyre2. (**b**) Using Phyre2, it was observed that the Glu residue (109) would be involved in binding to the Zn of the catalytic site, as highlighted in red. (**c**) Through p2rank, a better visualization of the residues involved in the active site was observed, and the amino acids involved are indicated with a solid line.

The three-dimensional (3D) structure was predicted to identify the active site of the 110-Mh metalloprotease. The 3D modeling prediction was performed through the 1Q2LA template of the crystallized *Escherichia coli* protein with 37.1% coverage and 44.3% score (Figure 6a). The Zn atoms of the catalytic site are possibly linked to glutamate (109), as suggested by the results obtained (Figure 6b). The possible active site is located where the histidine (106 and 110) and glutamate (109) residues are coupled to a H_2_O molecule (Figure 6c).

### 2.8. Anti-110-Mh Metalloprotease Antibodies Inhibit Its Proteolytic Activity

**Figure 7:**
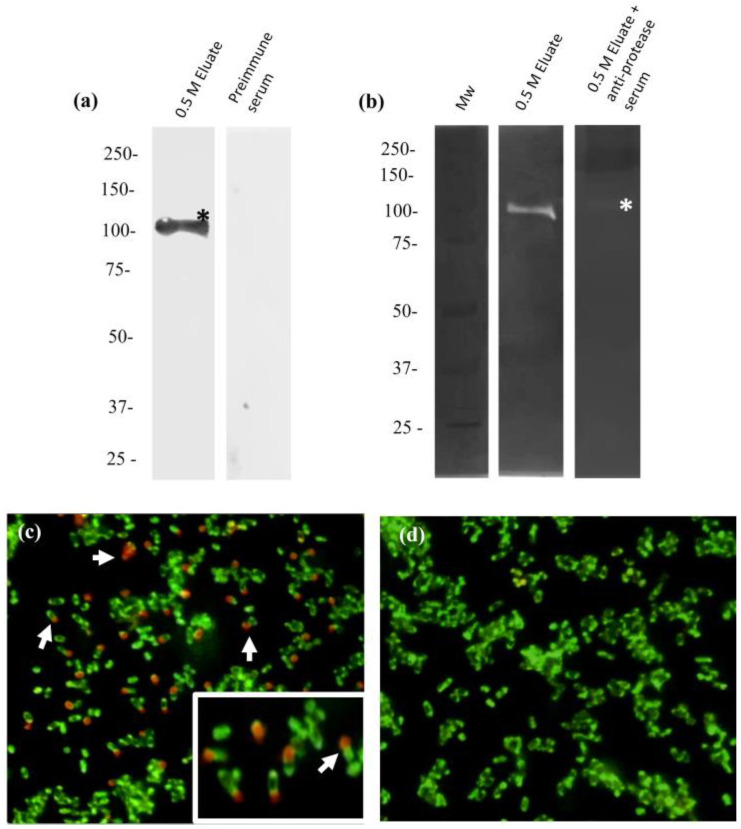
Inhibition and immunodetection of the 110-Mh metalloprotease. (**a**) Western blot of the 110-Mh metalloprotease. A single band was detected, indicated with an *. The assay with pre-immune serum is shown with the samples mentioned above as experimental controls. (**b**) Zymogram of gels copolymerized with 0.2% bovine collagen type 1; 110-Mh metalloprotease without incubation with the antiprotease serum was used as a control for proteolytic activity and 110-Mh metalloprotease preincubated with serum. Collagenolytic activity was almost completely inhibited, indicated with an *. (**c**) Immunofluorescence of the protease in smears of *M. haemolytica* A2. The arrows indicate the detection characterized by a red color, 200×. The insert shows a magnification of the image, 1500×. (**d**) Tests were carried out with pre-immune serum as an experimental control. The microphotographs were taken with a fluorescence microscope (Microphot-FXA, Nikon, Tokyo, Japan) with a DXM1200F microscope camera attached. Representative results of three independent sample trials.

Subsequently, to predict the catalytic site and model the 110-Mh metalloprotease, we produced anti-110 kDa Mh metalloprotease serum (Figure 7a). Western blot assays showed the presence of a 110 kDa protein in the eluate samples of 0.5 M NaCl (Figure 7a, Lane 1) from the CS of *M. haemolytica*; this protein was not observed using the pre-immune serum (Figure 7a, Lane 2). Moreover, we carried out immunodetection on a smear of bacteria through immunofluorescence. As seen in Figure 7c, detection of the 110-kDa Mh metalloprotease was observed in the smears of *M. haemolytica* A2. This result was mainly observed at the poles of the bacteria; in contrast, the pre-immune serum did not show a positive label (Figure 7d).

After the specific reaction among the antibodies was detected, inhibitory assays were performed. The incubation with the serum fraction that contains antibodies against the 110-Mh metalloprotease showed a great reduction in proteolytic activity in zymograms of gels copolymerized with bovine collagen type 1 (Figure 7b, Lane 3).

### 2.9. Identification of M. haemolytica A2 from a Suggestive Case of Mannheimiosis in Sheep through Histopathology and PCR

We obtained a biological sample of lungs with fibrinopurulent pleurobronchopneumonia (Supplementary Figure S1), and tissue samples from the lung lesions were processed. The analysis of microscopic lesions through histopathological sections stained with hematoxylin and eosin showed different morphopathological disorders compatible with *M. haemolytica*, such as dilated blood vessels full of erythrocytes and their extravasation, the presence of a homogeneous eosinophilic substance in alveolar spaces (congestion, hemorrhage, and edema), abundant inflammatory cells, mainly neutrophils in the alveoli, bronchioles, and pleura, and marked interseptal and pleural fibrinous exudate, in addition to multiple areas of liquefactive necrosis of bronchioles and alveolar epithelium (Supplementary Figure S2). Moreover, we found other areas of the lung that showed syncytia of macrophages, thickening of alveoli, and infiltration and proliferation of mononuclear inflammatory cells and hyaline membranes, suggesting interstitial pneumonia (Supplementary Figure S3).

**Figure 8:**
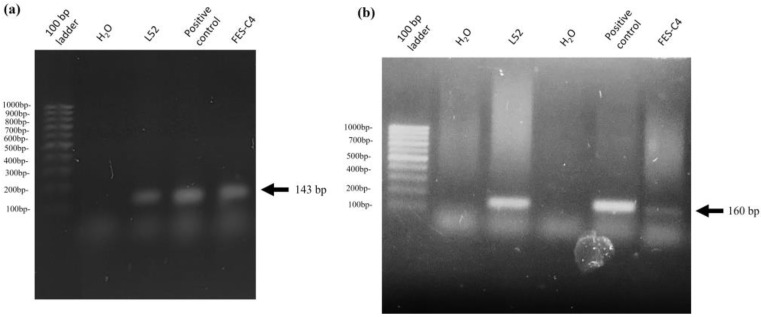
PCR. Electrophoresis was performed for 30 min to visualize the amplification products. (**a**) Agarose gel (1.5%) of the endpoint PCR of the *sod*A gene (143 bp) that determines *M. haemolytica*; sterile water and strain *M. haemolytica* were used as experimental controls. (**b**) Agarose gel of the endpoint PCR of the core-2/I-branching enzyme-encoding gene (160 bp) that determines serotype 2 of *M. haemolytica*; sterile water and strain *M. haemolytica* A2 were used as experimental controls. Amplifications of products of 143 bp and 160 bp were detected in both bacteria and are indicated by an arrow. Representative results of three independent samples.

Bacterial isolation and purification from the mentioned pneumonic lungs were performed. Microbiological identification was carried out through API 20 E tests and PCR. The data obtained were compared with those of the L52 strain used in the present study. The results showed that bacteria isolated (FESC-4 strain) from the lungs with pleurobronchopneumonia correlated with *M. haemolytica* (Figure 8a) belonging to serotype A2 (Figure 8b).

### 2.10. Detection of the 110-Mh Metalloprotease in Lung Samples from Sheep with Suggestive Pneumonic Damage of M. haemolytica

**Figure 9:**
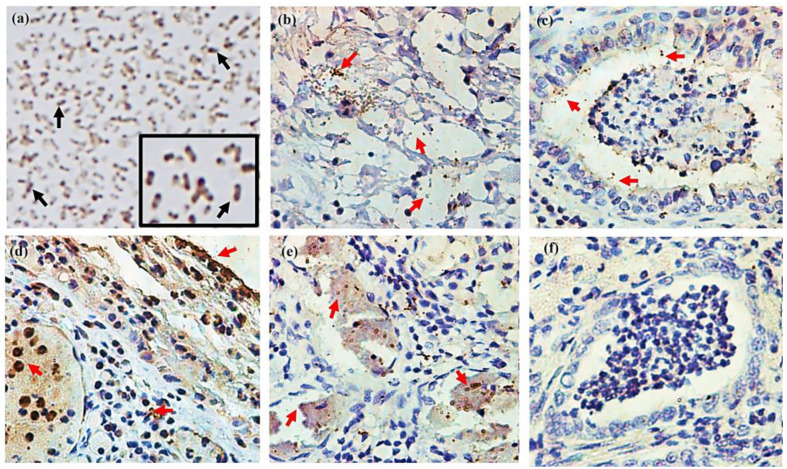
Immunohistochemistry of the 110-Mh metalloprotease. (**a**) Immunodetection of the 110-Mh metalloprotease in smears of *M. haemolytica* A2. The arrows indicate the protease characterized by a brown color (200×). The insert shows a magnification of the image (1500×). (**b**–**e**) Immunohistochemistry of 110-Mh metalloprotease from histopathological samples of lung tissue with fibrinopurulent pleurobronchopneumonia from which *M haemolytica* A2 was isolated (100×); 110-Mh metalloprotease was observed in the different morphopathological lesions: (**b**) fibrinous exudate, (**c**) bronchitis, (**d**) pleuritis, and (**e**) liquefactive necrosis. The red arrows indicate detection in the different lesions. (**f**) Tests were carried out with pre-immune serum as an experimental control. The microphotographs were taken with a fluorescence microscope (Microphot-FXA, Nikon, Tokyo, Japan) with a DXM1200F microscope camera attached. Representative results of three independent sample trials.

To determine whether the 110-Mh metalloprotease participates in the pathological process caused by *M. haemolytica*, lung tissues from the sheep previously mentioned were used. Anti-110-Mh metalloprotease antibodies were used on a smear of the isolated bacteria, and 110-Mh metalloprotease was observed (Figure 9a). The positive label was mainly observed at the poles of the bacteria, as correlated with our aforementioned findings. On the other hand, the immunohistochemistry analysis showed the 110-Mh metalloprotease close to the areas of suppurative lesions. The 110-Mh metalloprotease was observed in different morphopathological lesions, fibrinous exudate, bronchitis, pleuritis, and liquefactive necrosis (Figure 9b–e).

## 3. Discussion

*M. haemolytica* is the bacterial species associated with the respiratory disease complex in ruminants. This bacterium resides in the upper respiratory tract of healthy ruminants, although *M. haemolytica* can descend into the lungs in immunocompromised animals, such as those with a preexisting viral infection, leading to pneumonia [21]. During logarithmic growth, this bacterium produces and releases a potent leukotoxin (Lkt) [22] and an enzyme termed O-sialoglycoprotease (Gcp) [20]. LPS and Lkt have been implicated in initiating an inflammatory response in cattle [23,24,25,26]; however, it has not been demonstrated whether *M. haemolytica* proteases could intervene or participate in the lesions of mannheimioisis. Therefore, we evaluated the presence of proteolytic activities of the CS proteins of *M. haemolytica* A2. Through utilizing ammonium sulfate, we obtained different fractions containing distinct proteolytic activities. Proteases of 75, 110, and 250 kDa were found through zymography in gels copolymerized with bovine gelatin. The results revealed that most proteolytic activities were observed in the fraction with 60% ammonium sulfate saturation and were activated at pH 7.0. These results coincide with those reported in 2017 [17]. However, in that work, CS proteins were precipitated with ethanol, which could explain why 75 kDa activity was not observed before. Perhaps this activity with the protein precipitation method causes a change in the structure and loss of cleaving activity [27].

As part of the biochemical characterization, we evaluated the potential of proteolytic activities to degrade proteins, such as bovine holo-Lf, albumin, hemoglobin, and fibrinogen. We found that high molecular weight proteases (250 and >250 kDa) are nonspecific, promoting the cleavage of several proteins (hemoglobin, albumin, holo-Lf, and fibrinogen). These activities may play a relevant role in pathogenesis. Hemoglobin, albumin, and fibrinogen participate in intravascular oncotic pressure; thus, their degradation could promote edema, which is a finding in lung disease [11]. Degradation of holo-Lf could be implicated in the survival of the bacteria during infection, as previously described [15,28]. These proteolytic activities coincide with the proteases that degrade apo-Lf, which is the iron-free form of lactoferrin. We reported apo-Lf degradation in 2021, in which proteolytic activity was evaluated by zymography with CS proteins from *M. haemolytica* A2 [29]. Additionally, its degradation could be related to iron acquisition, and the release of iron after the proteolytic cleavage of holo-Lf could contribute to the growth of other microorganisms involved in the pneumonic respiratory complex that contains *M. haemolytica* [30], since Samaniego in 2016 reported that *M. haemolytica* does not use holo-Lf as an iron source [15]. On the other hand, the degradation of holo-Lf by proteases could generate small peptides of Lf named lactoferricins, which are more active than the parental molecule and exhibit antimicrobial activity toward this bacterium [31]. Degradation of fibrinogen may promote circulatory disorders, such as hemorrhage and edema. LPS could initiate vascular damage, and *M. haemolytica* could immediately or simultaneously secrete the 110-kDa protease to inhibit coagulation processes (hemostasis) and deposit in the interseptal and pleural spaces; this process could also result from the formation of fibrinopeptides, promoting neutrophil chemotaxis (inflammatory exudate) and morphopathological lesions characteristic of mannheimiosis [11,32].

On the other hand, only one proteolytic activity of 110 kDa selectively degraded gelatin. Gelatin is a collagen hydrolysate, and we used type-1 bovine collagen in our zymography assays and degradation kinetics to confirm our observations. A unique band of proteolytic activity was found for the 110 kDa protease. Furthermore, in the degradation kinetics results, we observed peptides derived from the alpha, beta, and gamma helices of collagen after incubation with the 110 kDa protease, indicating that the protease is a collagenase. We also wanted to test whether this protease could be heat-resistant, a multimeric protein, or a protein complex, as was reported for the protease secreted by *Actinobacillus pleuropneumoniae* by Negrete et al. in 1998 [33]. Our proteolytic activity was thermoresistant, and its activity was maintained even under treatment with reducing and chaotropic agents. Moreover, only phenanthroline, an inactivator of Zn metalloproteases, promoted inhibition of the 110 kDa activity [34]. Similar inhibition of collagenolytic activity was reported by Jackson et al. in 1997. In that study, collagenolytic activity was completely abolished using the synthetic peptide FALGPA, which is similar in structure to collagen [35]. In 2017, we reported that phenanthroline inhibited the activity of 100 kDa from culture supernatants of *M. haemolytica* A2 using porcine gelatin as a substrate [17].

Mass spectrometry is usually used to identify proteases [36]. In this work, the fingerprints suggested that a 110 kDa Zn-metalloprotease corresponding to protease 3 OS showed high levels of coverage and score within the *M. haemolytica* genome. Additionally, a potentially identified Zn-binding site corresponded to the sequence HYLEH (106–110); this sequence showed homology with the HEXXH motif of the zincin family, to which *Pseudomonas aeruginosa* elastase and metalloproteases from *A pleuropneumoniae*, *Legionella pneumophila*, *Vibrio cholerae*, and *V. vulnificus* belong [37,38]. Furthermore, the *M. haemolytica* A2 metalloprotease amino acid sequence showed high homology with other sequenced or predicted peptidases from several Gram-negative bacteria belonging to the Pasteurellaceae family, including *Haemophilus parasuis*, *A. pleuropneumoniae*, *Actinobacillus suis*, and *Bibersteinia trehalosi*.

Detection of the 110 kDa-Mh metalloprotease through Western blot, immunolocalization, and inhibitory assays was performed using antibodies. The inhibition of activity with antibodies is important because the severity of pneumonic lesions has been reduced with anti-Gcp vaccine trials. Shewen et al. in 2013 achieved protection through vaccination with a recombinant protein-Gcp in calves challenged with *M. haemolytica* A1. Even though the protective effect was greater than that of the combination of LKT with recombinant protein-Gcp or when only recombinant LKT was used; in this case, it was ineffective in reducing the severity of pneumonia after challenge [39,40]. Similar results were reported by McNeil et al. in 2003. In that study, the protease activity was neutralized by bighorn sera in a titratable manner. Furthermore, less necrotic tissue was observed at necropsy in animals challenged with *B. threalosi* (an isolate from a pneumonic bighorn sheep) compared to the control group [41]. These results demonstrate that, although LKT is recognized as the main virulence factor, the anti-LKT antibodies are not sufficient to protect against the disease.

Additionally, we had access to lung samples in which the isolation of *M. haemolytica* A2 was confirmed through PCR. Moreover, the detection of protease by immunohistochemistry showed the presence of bacteria and protease in the lung lesions of sheep with mannheimiosis. Identification of the secretion of IgG-degrading proteases by *M. haemolytica* in vivo has been reported. In 2020, Ayalew et al. sequenced and cloned two IgA1 and IgA2 peptidases with molecular weights of 96.5 and 87 kDa from *M. haemolytica* serotype A1. In addition, they observed a significant increase in serum antibodies against peptidase IgA1 and peptidase IgA2 in beef cattle recovered from respiratory illness, compared to healthy cattle with no history of illness [18]. Similar results were found for other bacteria of the Pasteurellaceae family. In a study, a protease (>200 kDa) from *A. pleuropneumoniae* was purified, and by immunoblotting, the protease was recognized by the serum of pigs naturally infected with serotypes 1 and 5 and by the serum of pigs experimentally infected with serotypes 1, 2, 8, or 9. This protease is expressed in vivo [33]. On the other hand, the detection of the protease in injured lung tissues was also reported by García González O. et al. in 2004 [38]. In that work, a metalloprotease secreted by *A. pleuropneumoniae* serotype 1 was purified. The immune reaction against the purified protease in porcine pleuropneumoniae-infected lung tissues was detected with antibodies, but this was not found in healthy lungs, indicating that protease expression occurred during the disease stages.

In other bacteria, the presence of collagenases has been reported, such as *Clostridium histolyticum* [42,43], *Clostridium perfringens* [44], *Streptococcus mutans* [35], *Borrelia burgdorferi* [45], *Vibrio parahaemolyticus* [46], *Fusobacterium nucleatum* [47], and, importantly, in *A. actinomycetemcomitans* [48] and *B. trehalose* [41]; these are bacterial genera belonging to the Pasteurellaceae family to which *M. haemolytica* belongs. It has been reported that these collagenases intervene in necrotic processes [49], act as virulence factors for invasiveness and tissue injury [50], and play important roles in invasiveness and transmission during infection. Importantly, the lungs are composed of a high percentage of collagen [51,52]. The extracellular matrix of the alveoli is composed of a relaxed meshwork, largely based on type I and III collagens and elastin as important core proteins [53]. In this way, collagenase could cause the destruction of lung tissue, establishing colonization and increased inflammation, worsening the disease process. Moreover, the 110-Mh metalloprotease could stimulate gelatinase production and release by leukocytes and is involved in neutrophil-associated gelatinase activation. According to the Starr et al. study in 2004, the CS products of *M. haemolytica* increase the levels of the matrix metalloproteinase gelatinases A and B in injured lung tissues; in addition, the products activate gelatinase B in neutrophils and cause the concentration of this enzyme to increase but not its activation in bovine monocytes. These results were similar when recombinant sialoglycoprotease were used in the same cells [54]. Although many biomolecules are secreted in the CS of *M. haemolytica*, it would be interesting to further evaluate whether the 110-Mh metalloprotease can induce and activate matrix metalloproteinase gelatinases from the host.

## 4. Materials and Methods

### 4.1. Bacterial Strains

The experimental tests were carried out with a field strain of *M. haemolytica* A2 (L52 strain) obtained from pneumonic lungs of a lamb that died from mannheimiosis; this strain has been used in previous works [17]. The confirmation was performed using biochemical methods: API 20 E (bioMérieux, Mexico City, Mexico) and PCR. Bacteria were grown on blood-agar plates for 24 h at 37 °C before the experiments.

### 4.2. Precipitation of Proteins from CS with (NH_4_)_2_SO_4_

To obtain the proteins from CS, bacterial growth was carried out by transferring a colony from a pure culture to 5 mL of brain–heart infusion (BHI) medium (Dibico, Mexico City, Mexico); the culture was incubated for 24 h at 37 °C. Then, 500 μL (1.98 × 10^7^ CFU) of the culture was transferred to 100 mL of BHI medium for 24 h at 37 °C. Subsequently, the cultures were centrifuged at 2600× *g* for 20 min, and the CS was filtered through a 0.22 µm pore diameter mixed cellulose ester membrane (Millipore, Dublin, Ireland) [29]. Afterward, proteins of CS were precipitated with (NH_4_)_2_SO_4_ (Sigma-Aldrich, St. Louis, MO, USA). The filtered CS was mixed with different concentrations of (NH_4_)_2_SO_4_; established saturation concentrations with (NH_4_)_2_SO_4_ were used for this procedure [55]. An initial saturation of 60% of (NH_4_)_2_SO_4_ was used with the CS proteins, which were subsequently centrifuged at 10,000× *g* for 1 h at 4 °C; the precipitate was resuspended with phosphate-buffered saline (PBS) 1X, the soluble fraction was recovered, and (NH_4_)_2_SO_4_ was added until a saturation of 70% was reached. This solution was centrifuged, and the precipitation of the soluble fractions continued at 80%. The pellet of each precipitate was resuspended in 500 μL of 1X PBS. Additionally, the samples were centrifuged on 30 kDa cutoff Amicon columns (Millipore) or dialyzed with PBS 1X for 12 h to eliminate the (NH_4_)_2_SO_4_. The samples obtained corresponded to proteins precipitated with (NH_4_)_2_SO_4_ (PpAs). The concentration of PpAs was determined by colorimetry using the Bradford microtiter method and bovine serum albumin (BSA) as a standard [56].

### 4.3. Zymography Assays of the PpAs from CS of M. haemolytica A2

The proteolytic activities were determined in 10% polyacrylamide gels copolymerized with 0.2% bovine gelatin (Sigma-Aldrich), holo-Lf (NutriScience Innovations, LLC, Milford, CT, USA), hemoglobin, albumin, and porcine fibrinogen (Sigma-Aldrich). The protein concentration of PpAs was adjusted to 20 µg for use in the electrophoretic run. Electrophoresis was performed at a constant voltage (100 V) for 2 h in an ice bath (4 °C). Samples were not treated with β-mercaptoethanol or boiled. After electrophoresis, the gels were washed twice with 2.5% (*v*/*v*) Triton X-100 (Sigma-Aldrich) solution for 30 min and incubated overnight at 37 °C with different buffers: 100 mM sodium acetate, 2 mM CaCl_2_ (pH 5.0), 100 mM Tris-2 mM CaCl_2_ (pH 7.0), and 100 mM Na_2_CO_3_-NaHCO_3_-2 mM CaCl_2_ (pH 9.0) (all from Sigma-Aldrich). Finally, the gels were stained with 0.5% (*w*/*v*) Coomassie brilliant blue R-250 (Bio-Rad, Feldkirchen, Germany). Protease activities were identified as clear bands on a blue background. The gels were subjected to a washing solution until the proteolytic bands were visualized. All assays were performed in three independent experiments.

### 4.4. Purification of the 110 kDa Protease by Ion Exchange Chromatography

The PpAs were loaded onto cation-exchange chromatography resin Hi-S cartridges (Bio-Rad), previously equilibrated with buffer with 20 mM tricine (Sigma-Aldrich), pH 8.5. To promote ionic interactions of proteins, the columns were left in oscillation at 4 °C overnight. Elution was performed using a discontinuous gradient of tricine containing 0.1, 0.2, 0.3, 0.4, and 0.5 M NaCl (1 mL/min). The eluates were passed through Amicon columns with a cutoff of 100 kDa (Millipore) to remove NaCl from the samples and concentrate the protein. The protein concentration was quantified by the Bradford method [56]. To determine the fraction in which the 110 kDa protease was present, zymograms copolymerized with bovine gelatin were made, and the proteolytic activity of each eluate was evaluated. In addition, a comparison of the protein profile by SDS-PAGE of the 60% PpAs with the eluate that showed the purified activity of 110 kDa was carried out [36]. All assays were performed in three independent experiments.

### 4.5. Proteolytic Degradation of Bovine Collagen Type I by the 110 kDa Protease of M. haemolytica A2

To determine whether the 110 kDa protease can degrade collagen and function as a collagenase-specific substrate protease, zymography was performed in gels copolymerized with 0.2% bovine collagen type I (Thermo Fisher Scientific, Waltham, MA, USA). In addition, an interaction was carried out between chaotropic and reducing denaturing agents with the 110 kDa protease prior to the electrophoretic run. Briefly, the following incubations were performed: 10 µg of the 110 kDa protease with 8 M urea, 10 µg of the 110 kDa protease with 10% β-mercaptoethanol for 1 h at 22 °C, or 110 kDa protease subjected to 100 °C for 5 min (all the reagents were purchased from Sigma-Aldrich). Additionally, combinations of the agents were used [33].

In addition, proteolytic degradation kinetics were determined through 10% SDS-PAGE under nonreducing conditions. For this assay, an incubation of 10 µg of the 110 kDa protease with 15 µg of bovine type-I collagen (Thermo Fisher Scientific) was carried out for 1, 6, and 12 h at 37 °C in 100 mM Tris–2 mM CaCl_2_ (pH 7.0). After incubation, electrophoresis was performed at a constant voltage (100 V) for 2 h in an ice bath (4 °C). The buffer pH 7 was incubated with bovine type-I collagen (Thermo Fisher Scientific) as a negative control [57]. All assays were performed in three independent experiments.

### 4.6. Characterization of the Proteolytic Activity of 110 kDa by Using Protease Inhibitors

For the proteinase inhibition assays, the 110 kDa protease was preincubated for 1 h at 22 °C with different inhibitors under constant agitation. The concentration of inhibitors was as follows: for cysteine proteases, 10 mM p-hydroxymercuribenzoate (pHMB), for serine and cysteine proteases, 5 mM phenylmethylsulfonyl fluoride (PMSF), and 5 mM EGTA, 5 mM EDTA, or 10 mM phenanthroline for metalloproteases (all inhibitors were purchased from Sigma-Aldrich). We loaded the samples onto 10% SDS-PAGE copolymerized with 0.2% bovine type-I collagen (Thermo Fisher Scientific) and then performed electrophoresis at 4 °C in an ice bath at 100 V for 2 h. The gels were then washed twice with 2.5% (*v*/*v*) Triton X-100 solution (Sigma-Aldrich) for 30 min, incubated overnight with 100 mM Tris-2 mM CaCl_2_ (pH 7.0), and then stained as mentioned before. All assays were performed in three independent experiments [17].

### 4.7. Preparation of Sample for LC-MS/MS Mass Spectrometry

Mass spectrometry was used to identify the Zn-dependent metalloprotease that showed collagenase activity from *M. haemolytica* A2 (110-Mh metalloprotease). The purified protein of 110 kDa was obtained from its band of zymogram and from SDS-PAGE to proceed with the in-gel digestion protocol. The selected bands were reduced with 10 mM dithiothreitol (DTT) (Sigma-Aldrich) in 50 mM NH_4_HCO_3_ (50 µL) for 30 min at 56 °C and alkylated with 55 mM iodoacetamide (IAA) (Sigma-Aldrich) for 30 min in darkness at room temperature in 50 mM NH_4_HCO_3_ (50 µL). The sample with the pieces of gel was dried down by a speed vac and then incubated at 37 °C overnight at 2.5 ng μL^−1^ trypsin (Promega, Fitchburg, WI, USA) in 50 mM ammonium bicarbonate (ABC). The next day, the extraction of peptides was performed using the following steps: 50 μL of 25% acetonitrile (ACN) (Sigma-Aldrich) was added to the gel for 10 min of sonication in a water bath, and 50 μL of 75% ACN was added to the gel. Both supernatants were mixed in a new tube. The tryptic peptides were dried in a vacuum centrifuge to proceed with desalting using ZipTip C18 (Millipore, Dublin, Ireland) following the manufacturer’s protocol [58].

### 4.8. LC-MS/MS Mass Spectrometry

The samples with 1 μg of total peptides were resolved on a Thermo Scientific Dionex UltiMate 3000 RSLC system using a PepSep 150 µm × 8 cm C18 column (PepSep, Marslev, Denmark) with 1.5 μm particle size (100 Å pores) heated to 40 °C. The peptides were eluted in a total run time of 60 min with a flow rate of 0.520 μL/min with mobile phases A (water/0.1% formic acid) and B (80% ACN/0.1% formic acid). The mobile phase B gradient increased from 3% to 30% over 55 min, followed by a 5 min step to 90% B. Peptides were analyzed on an Orbitrap Fusion Lumos (Thermo Fisher Scientific) mass spectrometer. The mass spectrometer was operated in data-dependent acquisition mode. A survey full scan MS (from *m*/*z* 375 to 1500) was acquired in the Orbitrap at a resolution of 60,000 (at 200 *m*/*z*). The ions with a charge state +2 to +7, found in the highest abundance, were selectively isolated in a cycle lasting 3 s. The isolation window had a width of 1.2 *m*/*z*. Subsequently, they were subjected to fragmentation using higher energy C-trap dissociation (HCD) with a normalized collision energy of 32% [58]. 

### 4.9. Database Search

The validation of peptide and protein identifications based on MS/MS was performed in a Sequest (Thermo Fisher Scientific; version IseNode in Proteome Discoverer 1.4.1.14) and X! Tandem (The GPM, thegpm.org; version X! Tandem Alanine, 2017.2.1.4 in Scaffold v5.1.2 Proteome Software Inc., Portland, Oregon) against the UniProtKB *M. haemolityca* database, 12,649 entries. If a peptide identification could be verified with a probability higher than 95.0%, it was deemed legitimate. Protein identifications with at least two identified peptides and a proven probability of greater than 99.0% were acceptable. We used the following search parameters: the digestion enzyme trypsin allows two missed cleavage; fragment ion mass tolerance of 0.60 Da; a parent ion tolerance of 20 PPM; carbamidomethylation of cysteine residues as fixed modification; oxidation of methionine residues deamidated of asparagine and glutamine as variable modification.

### 4.10. Prediction of the 3D Structure and Catalytic Site of the 110-Mh Metalloprotease

For analysis through mass spectrometry, a peptide fingerprint was selected based on a high identity value, molecular weight, and protease activity. Modeling, 3D visualization, and prediction of the active site were performed using Protein Homology/analogY Recognition Engine V 2.0 (Phyre 2, http://www.sbg.bio.ic.ac.uk/~phyre2/html/page.cgi?id=index, accessed on 10 March 2023) and PrankWeb: Ligand Binding Site Prediction (https://prankweb.cz/, accessed on 10 March 2023) [59,60].

### 4.11. Production of Anti-Protease Serum, Western Blotting, and Inhibition of 110-Mh Metalloprotease Activity by Antibodies

Antiprotease antibodies were produced as previously described with some modifications [61]. Briefly, the gel slice was rinsed in deionized water for a few minutes with several water changes and then placed on a piece of parafilm or transparent film. The gel was fragmented into small pieces with deionized water. New Zealand female rabbits were subcutaneously and intramuscularly immunized with 0.5 mg of protein from the fraction obtained previously emulsified with aluminum hydroxide to increase immunogenicity (*v*/*v*). Subsequently, the animals received two boosts of the same antigen at the same concentration over an interval of 10 days. To obtain serum, the animal was held, its ear was held, and the central artery was identified. Asepsis of the site was performed with 70% ethanol, and the puncture was performed by placing the needle at 10 degrees inclination with respect to the ear. Pre-immune (PI) serum was obtained before immunization. Later, to corroborate the production of antibodies present in the serum obtained, Western blot tests were performed. After electrophoretic separation by 10% SDS-PAGE, the proteins were transferred to a nitrocellulose membrane (Sigma-Aldrich) for 1 h at 400 mA according to Towbin et al. [62]. The membranes were blocked with skim milk for 2 h at 22 °C, and samples were incubated overnight with rabbit anti-protease serum (1:2000) at 4 °C. The samples were washed three times with PBS-Tween (0.05%) and incubated with a secondary anti-rabbit-HRP antibody (1:3000) (Santa Cruz, Santa Cruz, CA, USA) for 1 h at 22 °C. The membrane was washed seven times, and the signal was revealed with a luminol kit reagent (Sigma-Aldrich) using an Odyssey FC imaging system (LI-COR).

In addition, to determine whether the anti-protease serum could inhibit the 110-Mh metalloprotease activity, zymograms copolymerized with bovine type-1 collagen (Thermo Fisher Scientific) were loaded with 110-Mh metalloprotease (10 μg) and preincubated with the anti-protease serum (10 μg/mL) for 1 h at 22 °C. After incubation, electrophoresis was performed at a constant voltage (100 V) for 2 h in an ice bath (4 °C). The zymograms were activated overnight at pH 7.0 and 37 °C [36].

### 4.12. Determination and Characterization of M. haemolytica A2 Isolates from Lungs

The identification and characterization of the genus and species of the bacterial isolate from the pneumonic lungs (kindly donated by the diagnostic service of the Pathology Department of the FESC-UNAM, Mexico) was carried out through PCR, as previously described [63]. Briefly, DNA extraction was performed with the phenol-chloroform method [64], and the purity and concentration were measured using the COLIBRI Titertek Berthold LB 915 Micro-Volume Spectrophotometer (Berthold Technologies, Bad Wildbad, Germany). Multiplex PCR was performed to determine the genus and serotype for *M. haemolytica* isolate. AmpliTaq Gold TM Fast PCR Master Mix (Thermo Fisher Scientific) was used with the primers *M. haemolytica*, 5′-AGCAGCGACTACTCGTGTTGGTTCAG-3′ and 5′-AAGACTAAAATCGGATAGCCTGAAACGCCTG-3′, corresponding to the *sod*A gene of 143 bp. For serotype 2, Nucleo2_F 5′-GGCATATCCTAAAGCCGT-3′, Nucleo2_R 5′-AGAATCCACTATTGGGCACC-3′ corresponded to the core-2/I-Branching enzyme-encoding gene of 160 bp. The reaction was carried out in a Multigene Optimax Labnet thermocycler (Labnet, Edison, NJ, USA) under the following conditions: initial denaturation—95 °C × 15 min × 1 cycle; denaturation—94 °C × 30 s × 35 cycles; alignment—55 °C × 45 s × 35 cycles; extension—72 °C × 1 min × 35 cycles; final extension—72 °C × 10 min × 1 cycle. Finally, electrophoresis was performed in a 1.5% agarose gel for 30 min to visualize the reaction products. The results of bacterial isolate (FESC-4) from pneumonic lungs were compared with the strain used in this work (L52). The positive experimental control of *M. haemolytica* A2 is a field strain isolated from sheep lungs with mannheimiosis, kindly donated by National Center for Disciplinary Research in Animal Health and Safety, INIFAP, Mexico.

### 4.13. Histopathology, Immunohistochemistry, and Immunofluorescence

The lung lesions were selected and fixed with 4% formalin for 24 h. Samples were dehydrated with gradual concentrations of alcohol (60, 70, 80, 90, and 100%) (J.T. Baker, Phillipsburg, NJ, USA) and embedded in paraffin; next, 4 µm semi-fine sections were cut from the paraffin blocks for hematoxylin and eosin (H&E) (Sigma-Aldrich) staining. Furthermore, to detect the protease, immunohistochemistry of the lung lesions was performed. For this, antigen retrieval was necessary; then, after dewaxing, the sections were immersed in 10 mmol/L citric acid (Sigma-Aldrich) pH 6. Subsequently, the slides were incubated in 0.9% H_2_O_2_ for 15 min and rinsed with PBS tween 1×. Afterward, rabbit anti-protease serum (1:100) was added and incubated for 1 h at 22 °C. The slides were washed with PBS-Tween 1X and incubated with a secondary anti-rabbit-HRP antibody (1:200) (Santa Cruz, Santa Cruz, CA, USA) for 1 h at 22 °C. Sections were rinsed with PBS-Tween 1X, and peroxidase activity was developed for 10 min with 0.7 mg/mL diaminobenzidine (Sigma-Aldrich).

For immunofluorescence, bacteria were labeled with fluorescein isothiocyanate (FITC) (Sigma-Aldrich) according to Dagmara et al. [65]. Overnight cultures of *M. haemolytica* were centrifuged and washed with PBS. Bacteria (2 × 10^9^) were added to 10 mL of 0.01% FITC (Sigma-Aldrich) in 0.2 M Na_2_CO_3_/NaHCO_3_ buffer, pH 9.6, and incubated on ice for 15 min. Bacterial cells were then washed and resuspended in RPMI medium (Gibco, New York, NY, USA) at a final concentration of 1 × 10^8^ CFU/mL. Bacteria were fixed for 1 h in 4% paraformaldehyde (Sigma-Aldrich) dissolved in PBS. After this, the cellular suspension was washed twice with PBS, blocked with 1% BSA at room temperature, and incubated at 37 °C for 1 h with anti-protease serum (1:100) and then with a rhodamine-labeled goat IgG-anti-rabbit antibody (1:200), (Sigma-Aldrich). Samples were poured on glass slides and observed by epifluorescence microscopy [38]. The microphotographs were taken with a fluorescence microscope (Microphot-FXA, Nikon, Tokyo, Japan) with a DXM1200F microscope camera attached. Representative results of three independent sample trials were derived.

## 5. Conclusions

For the first time, we identified and purified collagenase in pneumonic lung lesions caused by *M. haemolytica* A2. Our data strongly suggest that the 110-Mh metalloprotease participates as an important virulence factor in the pathogenesis of *M. haemolytica* and could participate in the disease progression and tissue damage by promoting necrotic lesions and inflammation. Furthermore, we observed an inhibition with anti-protease serum, so this protease is antigenic and could be used as a therapeutic target in combination with some other therapeutic alternative to treat the disease.

## Data Availability

Data contained within the article.

## Supplementary Materials

The following supporting information can be downloaded at: https://www.mdpi.com/article/10.3390/ijms25021289/s1.
