# Supplementary material for: Identification, Biochemical Characterization, and In Vivo Detection of a Zn-Metalloprotease with Collagenase Activity from Mannheimia haemolytica A2
Source: Int J Mol Sci. 2024 Jan 20;25(2):1289. doi: 10.3390/ijms25021289 (PMC10816954; doi:10.3390/ijms25021289)
Supplement: Supplementary file 1 [file ijms-25-01289-s001.zip › ijms-2788780-supplementary.pdf]

International Journal of *Molecular Sciences*

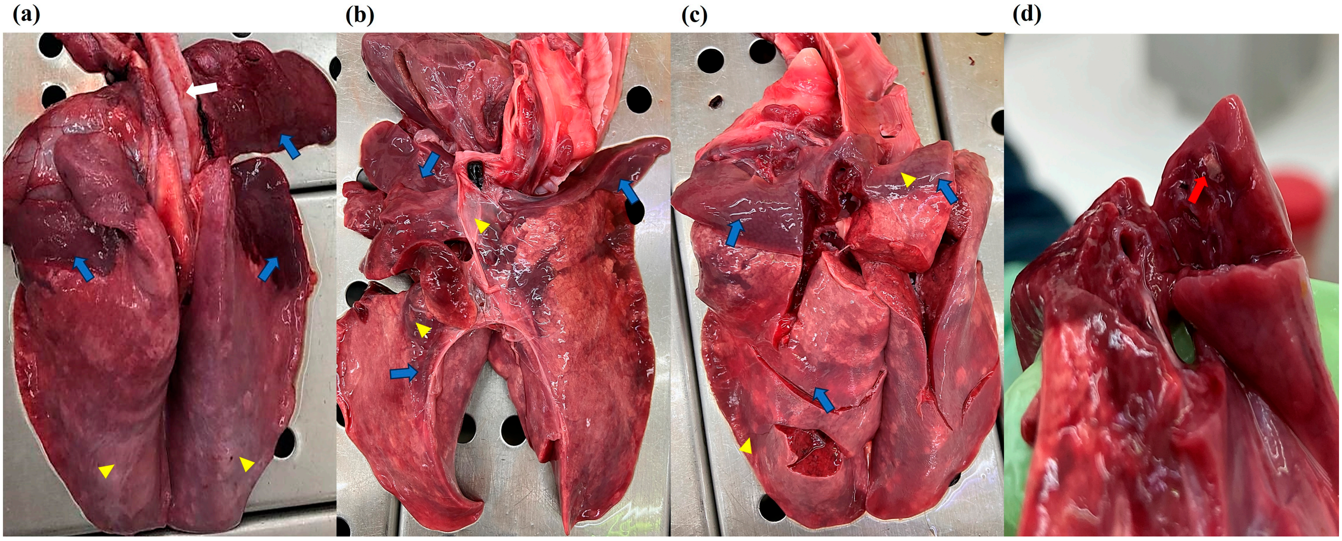

Figure S1. Macroscopic analysis of lungs from sheep with morphopathological disorders. (a-c) Lungs of small ruminants with different macroscopic findings compatible with pneumonia caused mainly by *M. haemolytica*: lung consolidation (blue arrow) and thickening of the lung pleura (yellow arrowheads). **(d)** Lung cut surface with purulent exudate (red arrow).

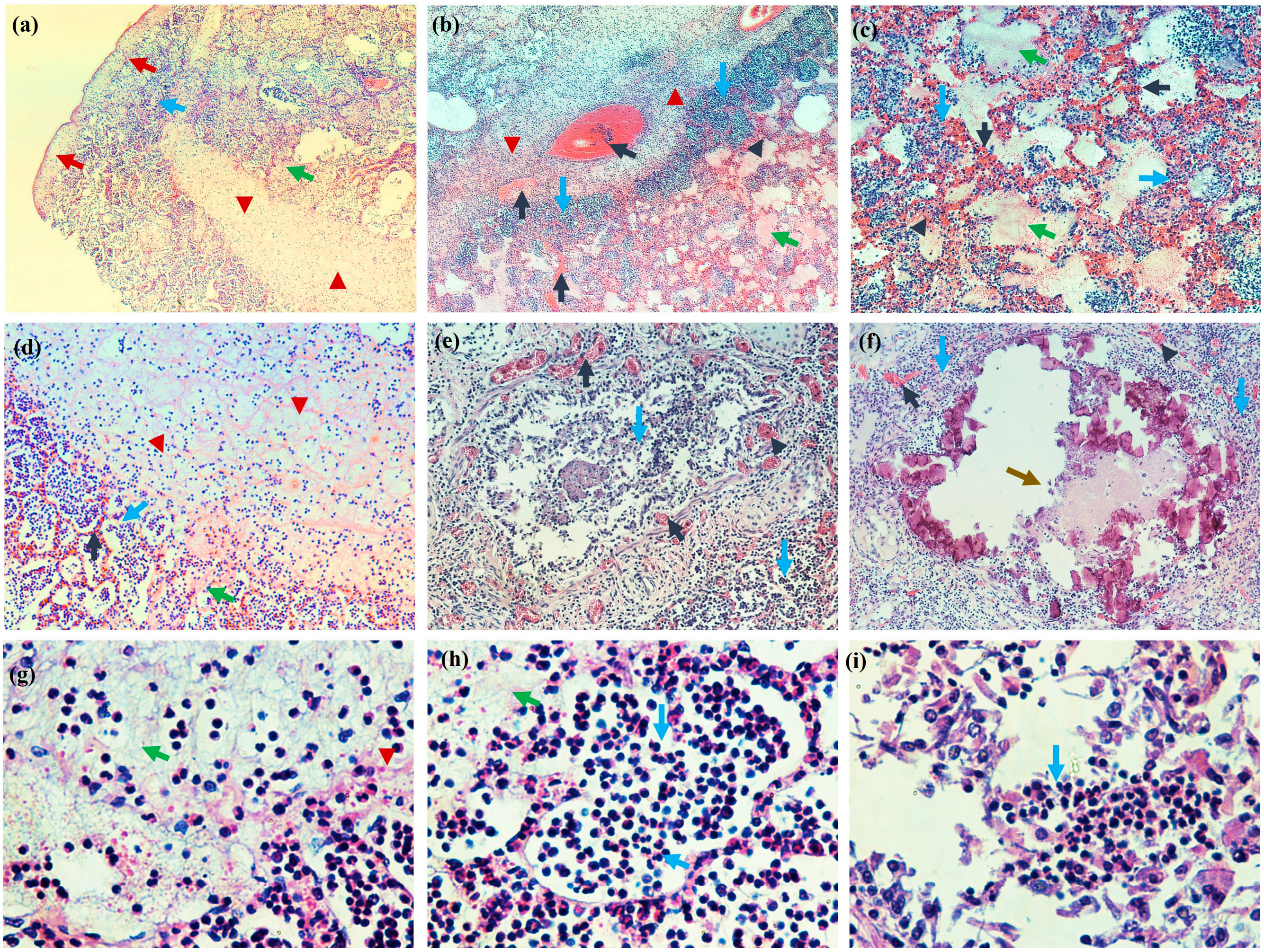

Figure S2. Histopathological disorders compatible with lesions caused *by M. haemolytica*. (a-d) Congestion (black arrows), hemorrhage (black arrowheads), edema (green arrows), pleural (red arrows) and interseptal (red arrows heads) fibrinous exudate, 100x. **(e and f)** Liquefactive necrosis (brown arrow), 100x. **(g-i)** Inflammatory infiltrate characterized mainly by neutrophils (blue arrows), 200x. The microphotographs were taken with a fluorescence microscope (Microphot-FXA, Nikon, Tokyo, Japan) with a DXM1200F microscope camera attached**.** Representative results of three independent sample trials.

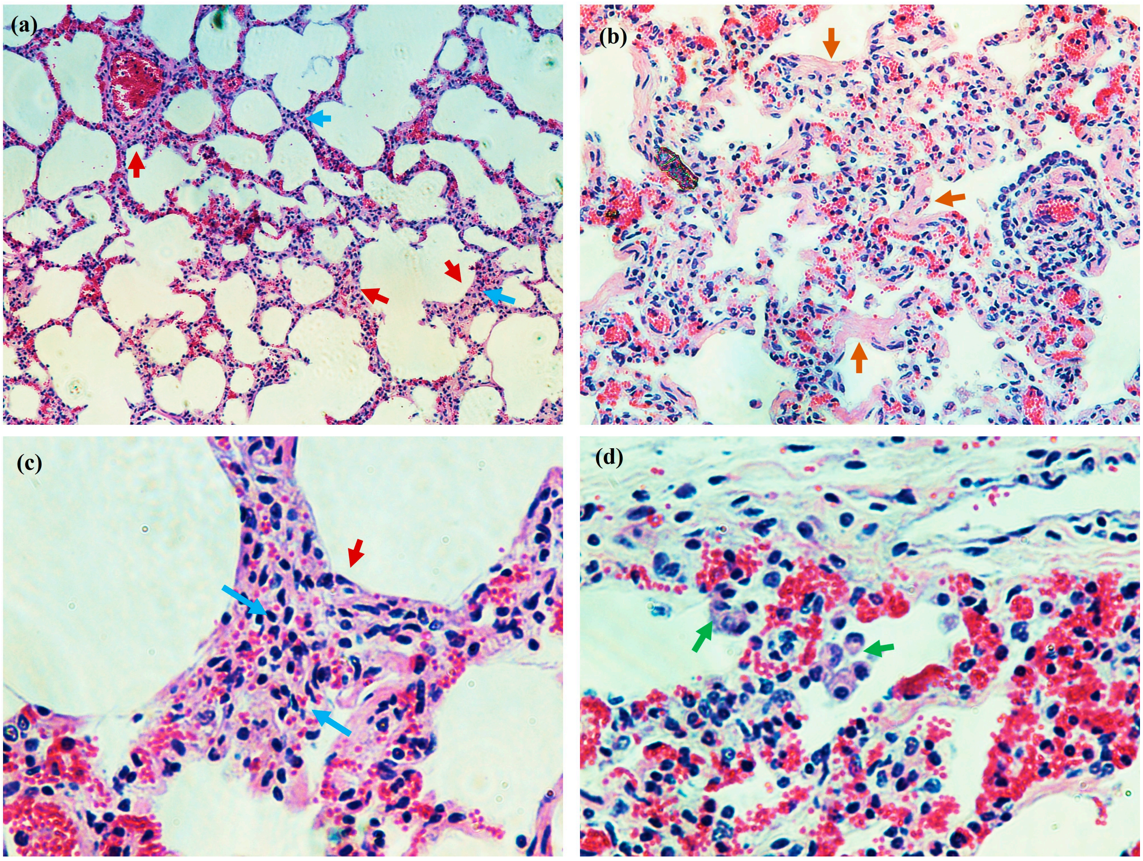

Figure S3. Morphopathological lesions compatible with etiologies of viral origin. (a) Thickening of alveoli (red arrows) and **(b)** hyaline membranes (orange arrows), 100x. **(c)** Infiltration and interstitial proliferation of mononuclear inflammatory cells (blue arrows). **(d)** Syncytia of macrophages (green arrows), 200x. The microphotographs were taken with a fluorescence microscope (Microphot-FXA, Nikon, Tokyo, Japan) with a DXM1200F microscope camera attached**.** Representative results of three independent sample trials.
